# Novel Computed Tomography Perfusion and Laboratory Indices as Predictors of Long-Term Outcome and Survival in Acute Ischemic Stroke

**DOI:** 10.3390/neurolint17090136

**Published:** 2025-08-27

**Authors:** Eray Halil, Kostadin Kostadinov, Nikoleta Traykova, Neli Atanasova, Kiril Atliev, Elizabet Dzhambazova, Penka Atanassova

**Affiliations:** 1 Department of Neurology, Medical University of Plovdiv, 15A Vasil Aprilov Blvd., 4002 Plovdiv, Bulgaria; 2 Clinic of Neurology, UMHAT “St George”, 66 Peshtersko Shose Blvd., 4001 Plovdiv, Bulgaria; 3 Department of Social Medicine and Public Health, Medical University of Plovdiv, 15A Vasil Aprilov Blvd., 4002 Plovdiv, Bulgaria; kostadinr.kostadinov@mu-plovdiv.bg (K.K.);; 4 Environmental Health Division, Research Institute at Medical University of Plovdiv, 15A Vasil Aprilov Blvd., 4002 Plovdiv, Bulgaria; 5 Department of Radiology, UMHAT “St George”, 66 Peshtersko Shose Blvd., 4001 Plovdiv, Bulgaria; nikoleta.traykova@mu-plovdiv.bg (N.T.);; 6 Strategic Research and Innovation Program for the Development of MU-PLOVDIV–(SRIPD-MUP), European Union-NextGeneration EU, BG-RRP-2.004-0007-C01, 4002 Plovdiv, Bulgaria; 7 Department of Emergency Medicine, UMHAT “St George”, 66 Peshtersko Shose Blvd., 4001 Plovdiv, Bulgaria

**Keywords:** acute ischemic stroke, CT perfusion, hypoperfusion intensity ratio, inflammatory markers, functional outcome, stroke prognosis, survival, thrombolysis

## Abstract

Background/Objectives: Acute ischemic stroke is a leading cause of mortality and long-term disability globally, with limited reliable early predictors of functional outcomes and survival. This study aimed to assess the prognostic value of two novel predictors: the hypoperfusion intensity ratio calculated from mean transit time and time-to-drain maps (HIR-MTT–TTD), derived from computed tomography perfusion (CTP) imaging parameters, and the Inflammation–Coagulation Index (ICI), which integrates systemic inflammatory (C-reactive protein and white blood cell count) and hemostatic (D-dimer) markers. Methods: This prospective, single-center observational study included 60 patients with acute ischemic stroke treated with intravenous thrombolysis and underwent pre-treatment CTP imaging. HIR-MTT–TTD evaluated collateral status and perfusion deficit severity, while ICI integrated C-reactive protein (CRP), white blood cell (WBC) count, and D-dimer levels. Functional outcomes were assessed using the National Institutes of Health Stroke Scale (NIHSS), Barthel Index, and modified Rankin Scale (mRS) at 24 h, 3 months, and 1 year. Results: Of 60 patients, 53.3% achieved functional independence (mRS 0–2) at 1 year. Unadjusted Cox models showed HIR-MTT–TTD (HR = 6.25, 95% CI: 1.48–26.30, *p* = 0.013) and ICI (HR = 1.08, 95% CI: 1.00–1.17, *p* = 0.052) were associated with higher 12-month mortality, worse mRS, and lower Barthel scores. After adjustment for age, BMI, smoking status, and sex, these associations became non-significant (HIR-MTT–TTD: HR = 2.83, 95% CI: 0.37–21.37, *p* = 0.314; ICI: HR = 1.07, 95% CI: 0.96–1.19, *p* = 0.211). Receiver operating characteristic (ROC) analysis indicated moderate predictive value, with ICI (AUC = 0.756, 95% CI: 0.600–0.867) outperforming HIR-MTT–TTD (AUC = 0.67, 95% CI: 0.48–0.83) for mortality prediction. Conclusions: The study introduces promising prognostic tools for functional outcomes. Elevated HIR-MTT–TTD and ICI values were independently associated with greater initial stroke severity, poorer functional recovery, and increased 1-year mortality. These findings underscore the prognostic significance of hypoperfusion intensity and systemic thrombo-inflammation in acute ischemic stroke. Combining the use of the presented indices may enhance early risk stratification and guide individualized treatment strategies.

## 1. Introduction

Stroke remains one of the greatest burdens to global health; it is the second leading cause of death worldwide and a leading cause of long-term disability [1]. Each year, approximately 15 million people suffer a stroke, of whom 5 million die and another 5 million are left permanently disabled [2]. Survivors often face significant neurological deficits, making stroke the primary cause of adult disability in many countries [1]. Given the enormous impact, improving acute stroke outcomes is a public health priority.

Advanced acute interventions, including intravenous thrombolysis and endovascular treatment, substantially improve outcomes when administered promptly. Nevertheless, patient outcomes remain highly variable even when treatments adhere to clinical guidelines [3].

There is a critical need for reliable prognostic tools to stratify patients based on expected recovery, guide treatment decisions, and facilitate discussions with patients and families regarding likely outcomes. Predicting functional recovery or mortality in acute ischemic stroke is complex, as it hinges on multiple factors, including the degree of brain ischemia, collateral circulation, reperfusion success, and systemic conditions [4,5]. This complexity has driven efforts to identify imaging biomarkers and clinical indices that can early in the stroke process indicate which patients are likely to have favorable outcomes and which face a higher risk of poor prognosis [6].

Recent evidence underscores the prognostic importance of systemic inflammatory and coagulation biomarkers in acute ischemic stroke. Elevated admission levels of C-reactive protein (CRP) have been repeatedly linked to poorer functional recovery and higher mortality, independent of established clinical predictors [7,8,9,10]. Similarly, meta-analyses and prospective cohort studies demonstrate that elevated D-dimer on admission is associated with increased risk of early recurrence, unfavorable functional outcome, and mortality at 30- and 90-day follow-ups [11,12]. These findings suggest that markers such as CRP and D-dimer reflect systemic thrombo-inflammatory activity that may interact with cerebral perfusion dynamics to influence recovery trajectories.

We hypothesized that two novel indices—the hypoperfusion intensity ratio derived from mean transit time and time-to-drain maps (HIR-MTT–TTD) and the Inflammation–Coagulation Index (ICI) would provide independent prognostic value for functional recovery and survival in patients with acute ischemic stroke treated with intravenous thrombolysis.

### 1.1. Evolving Role of Computed Tomography Perfusion (CTP) in Acute Ischemic Stroke

Over the past decade, advanced imaging, particularly CTP, has emerged as a critical tool in managing acute ischemic stroke. Unlike non-contrast computed tomography (CT), which provides a static anatomical view, CTP delivers real-time maps of cerebral blood flow, volume, and transit times [13]. This allows clinicians to distinguish irreversibly infarcted brain tissue (ischemic core) from hypoperfused but salvageable tissue at risk (penumbra) [14]. By delineating these regions, CTP can identify patients who have a significant penumbra despite an occluded artery, indicating that urgent reperfusion therapy could still salvage brain tissue even outside the traditional time window [15]. CTP has thus increasingly been used to guide treatment decisions, especially in scenarios where time-based criteria are insufficient [14]

Key clinical contexts where CTP imaging plays a pivotal role include wake-up strokes (WUS), strokes of unknown onset (SUKO), extended therapeutic window (ETW) presentations, and stroke mimics (SM) [16]. In WUS and SUKO cases, where the time of symptom onset is unclear or unwitnessed, CTP enables treatment decisions based on tissue viability rather than strict temporal criteria [17]. This tissue-based approach, supported by clinical trials [15], allows identification of salvageable penumbra and safe administration of reperfusion therapy. In ETW patients—those presenting beyond conventional time windows for intravenous thrombolysis or mechanical thrombectomy—CTP can identify “slow progressors” with preserved penumbral tissue, thereby expanding treatment eligibility [18]. Lastly, in suspected stroke mimics, which constitute up to 30% of acute stroke evaluations [19], CTP enhances diagnostic accuracy by differentiating true ischemic events from non-vascular causes through the presence or absence of perfusion deficits.

Recent landmark trials have solidified the role of perfusion imaging in stroke care. DEFUSE 3 [20], a randomized trial, demonstrated that mechanical thrombectomy up to 16 h after symptom onset significantly improved outcomes in selected patients, with 45% achieving mRS 0–2 versus 17% in the control group. Notably, 75% of the participants were selected based on CT perfusion imaging. DAWN [21] extended the thrombectomy window to 24 h, as 49% of the patients achieved good outcomes compared to 13% in controls. CT perfusion guided selection in 67% of cases. EXTEND [22] demonstrated that CT perfusion can safely guide intravenous thrombolysis up to 9 h after stroke onset. Among patients selected (99% via CTP), 35.4% achieved excellent outcomes (mRS 0–1) versus 29.5% with placebo, with no significant increase in mortality. Inouse et al. [18] found slower infarct growth (~0.6 mL/h) in late-presenting patients with penumbral imaging compared to ~5.1 mL/h in early presenters, highlighting biological variability and the importance of collateral circulation. Guidelines from ESO and AHA/ASA [23] now endorse CTP or diffusion-weighted magnetic resonance imaging (DWI-MRI) for selecting reperfusion candidates, shifting care from time-based to tissue-viability-based decisions, improving outcomes, but prompting new questions about imaging-based prognostication.

While CTP has significantly broadened the therapeutic landscape in acute ischemic stroke, its prognostic value remains contentious. A major challenge involves the reliability of perfusion-derived metrics, particularly ischemic core volume, which can vary across software platforms and threshold parameters [24]. Notably, CTP may overestimate the infarct core, especially when performed within the first hour post-onset. In such ultra-early timeframes, severely hypoperfused but potentially salvageable tissue may be misclassified as irreversibly damaged—a phenomenon referred to as the “ghost infarct core” [25]. This can result in unwarranted treatment exclusion or misleading prognostic conclusions. Studies indicate that in rapidly reperfused patients, final infarct volumes can be markedly smaller than initial CTP estimates, particularly when using standard relative cerebral blood flow (rCBF) thresholds (e.g., <30%) [25]. Stricter thresholds (e.g., rCBF < 20%) may improve accuracy in early presenters, though no consensus standard has been established [26].

Furthermore, CTP offers a static snapshot of cerebral perfusion and does not account for dynamic determinants of outcome, such as time-to-reperfusion, collateral status evaluation, or complications like hemorrhagic transformation [27]. Two patients with identical baseline perfusion patterns may experience vastly different clinical trajectories depending on subsequent treatment success and systemic factors. Consequently, while CTP enhances acute management, its utility in predicting long-term outcomes is limited unless integrated with broader clinical and temporal context [28].

Despite the broad clinical use of CTP in acute ischemic stroke, its value for long-term prognostication remains uncertain. Existing research rarely integrates perfusion-derived metrics, such as the hypoperfusion intensity ratio (HIR), with systemic inflammatory and coagulation markers, both of which influence stroke evolution and recovery. The interaction between cerebral hypoperfusion and thrombo-inflammation is insufficiently studied, limiting current prognostic models. This study addresses this gap by evaluating both CTP-based indices and the Inflammation–Coagulation Index (ICI) in patients treated with intravenous thrombolysis, aiming to improve individualized risk stratification.

### 1.2. Aim of the Study

To address limitations in current prognostic tools, this study evaluates two novel indices to improve outcome prediction in acute ischemic stroke. The Hypoperfusion Intensity Ratio–mean transit time and time-to-drain (HIR-MTT–TTD) combines mean transit time (MTT) and time-to-drain (TTD) to measure perfusion deficits, extending beyond the traditional hypoperfusion intensity ratio (HIR), which uses time-to-maximum (Tmax) thresholds, by capturing microvascular impairment. The Inflammation–Coagulation Index (ICI) reflects systemic inflammatory and hypercoagulable states.

The study assesses the ability of HIR-MTT–TTD and ICI to predict one-year functional outcomes (neurological recovery and independence) and mortality in patients who underwent intravenous thrombolysis.

## 2. Materials and Methods

### 2.1. Study Design and Setting

This prospective, single-center observational study was conducted from January 2020 to December 2023 at the Department of Neurology, University Hospital “St. George” in Plovdiv, Bulgaria, the largest tertiary referral center in Southern Bulgaria. The study enrolled 60 consecutive patients (30 males, 30 females) admitted to the emergency department with a confirmed diagnosis of acute ischemic stroke based on clinical evaluation and non-contrast computed tomography. All participants underwent CTP imaging prior to receiving intravenous thrombolysis with alteplase (0.9 mg/kg) within 4.5 h of symptom onset, per guideline recommendations, and were followed for 12 months.

The sample size was determined using Slovin’s formula, with a 10% margin of error and 95% confidence level, based on the previous year’s thrombolysis patient population (N = 150). Slovin’s formula was selected because it allows for an adequate sample estimation when population size is known, variability is uncertain, and detailed pilot data are unavailable. This approach ensured feasibility within the resource constraints of a prospective imaging-based study while maintaining acceptable statistical precision.

**Figure A1:**
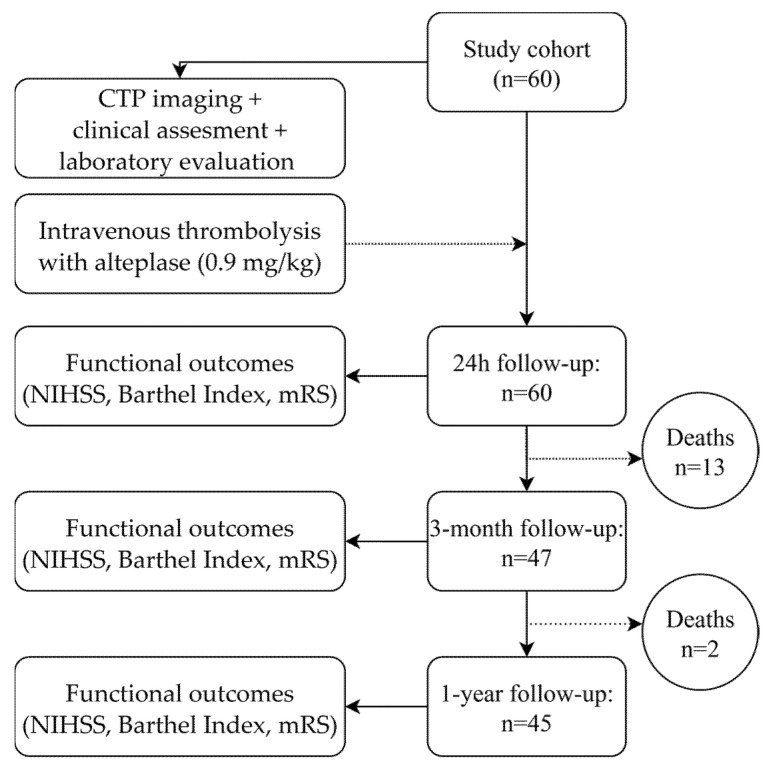
Flow diagram of patient follow-up.

Functional outcomes were assessed at 24 h, 3 months, and 1 year using the National Institutes of Health Stroke Scale (NIHSS) [29], Barthel Index [30], and modified Rankin Scale (mRS) [31]. If a patient died before a scheduled follow-up, the mRS score at the next time point was recorded as 6 (maximum value indicating death status), while NIHSS and Barthel Index were not imputed with extreme values; instead, only the last observed scores prior to death were retained (Appendix A, Figure A1). For analyses requiring complete trajectories, an additional variable was created to reflect the last known functional outcome score for each patient.

Unlike conventional prognostic approaches that rely solely on the NIHSS, which is based exclusively on the neurological examination, these indices provide complementary pathophysiological insights. HIR-MTT–TTD captures cerebral perfusion dynamics and microvascular impairment, while ICI reflects systemic inflammatory and hemostatic activity through relevant biomarkers. Together, they offer a more integrated and biologically informed prediction of patient outcomes.

Patients were eligible if they were aged ≥18 years, had a confirmed acute ischemic stroke, were suitable for intravenous thrombolysis (IVT), and had complete pre-treatment CTP imaging. Exclusion criteria included significant pre-existing neurological or cognitive impairment precluding accurate assessment, suspected or confirmed intracranial hemorrhage, contraindications to contrast-enhanced CT (e.g., allergy or renal impairment), lacunar infarction on imaging, and severe heart failure (New York Heart Association class III or higher).

Data were collected under ethical approval from the hospital’s institutional review board, and all participants or their legal representatives provided written informed consent.

### 2.2. Clinical and Demographic Data Collection

Demographic and clinical data were systematically collected through structured interviews and a comprehensive review of medical records. Collected variables included age, sex, and established vascular risk factors. Additional clinical information encompassed time from symptom onset to thrombolytic treatment, baseline blood pressure, and key laboratory parameters including C-reactive protein (CRP), white blood cell (WBC) count, and D-dimer levels, with elevated values defined as CRP > 10 mg/L, WBC > 10.5 × 10^9^/L, and D-dimer > 0.5 mg/L according to institutional standards.

To ensure consistency and reduce inter-rater variability, all evaluations were conducted by trained neurologists following standardized assessment protocols. Mortality data were also systematically recorded, including in-hospital deaths and deaths occurring during the 12-month follow-up period, verified through hospital records and the national death registry.

### 2.3. CT Perfusion Acquisition Protocol

CTP imaging was performed using a Siemens Somatom Definition AS 64-slice CT scanner (Siemens Healthineers, Erlangen, Germany) with syngo.via VB60 software for post-processing [32]. The scan was acquired in 4D spiral mode, capturing dynamic contrast passage through the brain over 45 s. Acquisition parameters included 80 kV tube voltage, 200 mAs current, 0.33 s rotation time, 5 mm slice thickness, and 1.5 s cycle time, covering a 62 mm scan range. Contrast medium (35–50 mL of non-ionic iodine, ≥300 mg I/mL) was injected at a rate ≥ 1.75 g/s, followed by a 20–30 mL saline flush.

Perfusion maps were automatically generated by the software, extracting key parameters: cerebral blood flow (CBF), cerebral blood volume (CBV), mean transit time, and time-to-maximum. These parameters enabled identification of ischemic core and penumbra.

### 2.4. Introduction of Novel Indices

To enhance the prognostic accuracy for acute ischemic stroke, we developed two novel composite indices to better capture hypoperfusion severity and systemic physiological states compared to conventional metrics. The HIR-MTT–TTD index was designed to quantify hypoperfusion severity and collateral circulation status with greater sensitivity than traditional Tmax-based approaches. This perfusion-derived metric integrates MTT and TTD delays, calculated using the formula:

$${\mathrm{HIR}}_{\mathrm{MTT}–\mathrm{TTD}}=\frac{\mathrm{Volume}\;\mathrm{MTT}>10s+\mathrm{Volume}\;\mathrm{TTD}>10s}{\mathrm{Volume}\;\mathrm{MTT}>6s+\mathrm{Volume}\;\mathrm{TTD}>6s}$$

A higher HIR-MTT–TTD value indicates more severe hypoperfusion and poorer collateral circulation. By combining MTT and TTD, this index captures microvascular impairment across multiple hemodynamic dimensions, offering improved sensitivity in detecting perfusion deficits compared to Tmax alone.

The thresholds of 6 s and 10 s for Tmax in the HIR-MTT–TTD formula were selected based on prior perfusion imaging literature and clinical practice in acute ischemic stroke. A Tmax delay > 6 s is widely accepted as indicative of hypoperfused but potentially salvageable penumbral tissue, while a delay > 10 s typically reflects severely hypoperfused core regions with markedly reduced collateral flow and poor prognosis. These cut-offs are consistent with thresholds used in major trials such as DEFUSE 3 [20]

The Inflammation–Coagulation Index was developed to integrate systemic inflammatory, coagulative, and immune responses, which are increasingly recognized as influencing stroke severity and recovery. The ICI is defined as:

(1) $$\mathrm{ICI}=\frac{\mathrm{CRP}+D-\mathrm{dimer}}{\mathrm{WBC}}$$

Here, CRP (mg/L) reflects systemic inflammation, D-dimer (µg/mL) indicates activation of the coagulation cascade, and WBC (×10^9^/L) represents immune reserve. A higher ICI suggests a pro-inflammatory and hypercoagulable state with a potentially compromised immune response, which may adversely affect stroke outcomes. Laboratory parameters were measured from blood samples collected at admission, prior to intravenous thrombolysis, using standardized assays to ensure consistency.

### 2.5. Statistical Analysis

Statistical analyses were conducted using R (version 4.3.2). Continuous variables, including HIR-MTT–TTD, Inflammation–Coagulation Index, cerebral blood flow, cerebral blood volume, mean transit time, time-to-maximum, time-to-drain, and ischemic core/penumbra volumes, were described using means ± standard deviations or medians (interquartile ranges) based on their distributional properties. Categorical variables, such as sex and vascular risk factors, were summarized as counts and percentages.

Missing data were handled using a complete-case approach for primary analyses. For functional outcomes (NIHSS, Barthel Index, mRS), if a patient died before a follow-up assessment, the mRS was coded as 6, and NIHSS/Barthel Index were assigned their last observed value; no imputation with extreme values was applied. For models requiring complete longitudinal data, a “last known score” variable was created to minimize attrition bias.

Correlation analyses were performed to explore associations between HIR-MTT–TTD, ICI, and functional outcome measures (NIHSS, Barthel Index, and mRS) at 24 h, 3 months, and 1 year. Multivariable Cox proportional hazards regression models were utilized to investigate the relationship between HIR-MTT–TTD and ICI with 12-month mortality, adjusting for potential confounders including age, sex, smoking status, and baseline clinical characteristics. Hazard ratios (HRs) with 95% confidence intervals (CIs) were computed, and model adequacy was evaluated using the Akaike Information Criterion (AIC) and likelihood ratio tests.

Discriminative performance for 12-month mortality was assessed via receiver operating characteristic (ROC) curves, with AUCs and 95% CIs calculated for both indices. ROC curves were smoothed using the binormal method to stabilize estimates and facilitate comparison. Optimal cut-offs were identified using Youden’s index, and sensitivity, specificity, and predictive values were reported.

To examine the associations between the key predictors—HIR-MTT–TTD and ICI— and long-term functional outcomes (mRS, NIHSS, and Barthel Index), a systematic framework of linear regression models was implemented. The outcomes were defined as the last known scores prior to death or censoring. For each outcome and predictor pair, three types of linear models were fitted: unadjusted models including only the predictor of interest; baseline-adjusted models additionally controlling for the corresponding baseline score (e.g., admission NIHSS); and fully adjusted models further accounting for age, sex, smoking status, and body mass index (BMI). Model results, including coefficients, standard errors, confidence intervals, and *p*-values, were extracted and visualized through forest plots with annotated effect estimates and 95% confidence intervals.

All tests were two-tailed, with *p* < 0.05 considered statistically significant. Analysis scripts used for model fitting, diagnostics, and figure generation are provided in the Supplementary Materials to support reproducibility.

## 3. Results

### 3.1. Baseline Characteristics

The study cohort comprised 60 patients (Table 1). The overall median age was 72 years (IQR: 62–79), with a significant difference observed between survivors and non-survivors (70 (60–76) vs. 85 (72–91) years, *p* < 0.01). The median BMI was 25.7 kg/m (IQR: 21.7–29.9), with higher values observed among survivors compared to non-survivors (26.8 (23.7–31.6) vs. 20.6 (18.6–25.7) kg/m, *p* < 0.01). Baseline systolic and diastolic blood pressures were comparable between groups (140 (120–160) mmHg and 80 (75–100) mmHg, respectively), with no statistically significant differences (*p* = 0.2 and *p* = 0.3).

Active smoking was reported in 40% of the study population, with similar proportions in both outcome groups (39% among survivors vs. 43% among non-survivors, *p* = 0.8). Comorbidities were common: diabetes was present in 73% of patients, and pre-existing cardiovascular disease (CVD) in 97%, with no significant differences by mortality outcome (*p* = 0.7 and *p* > 0.9, respectively).

Median MTT penumbra and infarct volumes were 20 mL (IQR: 14–25) and 5.0 mL (Interquartile range, IQR: 3.1–8.0), respectively, with a non-significant trend toward larger infarct volumes in non-survivors (6.3 [4.0–11.0] mL, *p* = 0.06). Similar findings were noted for TTD-based perfusion metrics.

Inflammatory and thrombotic markers showed significant differences: C-reactive protein and D-dimer levels were markedly elevated in non-survivors compared to survivors (CRP: 35 (16–56) vs. 10 (4–21) mg/L, *p* = 0.01; D-dimer: 4.7 [2.0–9.8] vs. 1.1 [0.5–3.2] mg/L fibrinogen equivalent units, *p* = 0.01). White blood cell (WBC) counts were similar between groups (*p* = 0.8).

### 3.2. Clinical Outcomes at 24 h, 3 Months, and 1 Year

**Figure 1:**
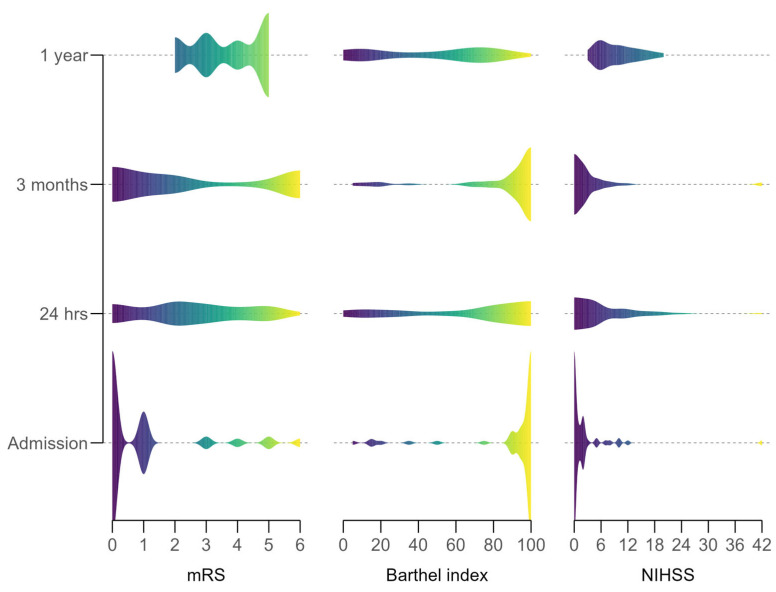
Distribution of neurological functional outcome scores over the study period.

Neurological and functional outcomes improved significantly over time post-thrombolysis (Figure 1). At 24 h, the median NIHSS score dropped to 5 (IQR 2–10.2) from 9 (IQR 6–13) at admission, reflecting early neurological improvement. The median mRS score decreased to 3 (IQR 2–4) from 4 (IQR 3–5), and the median Barthel Index rose to 80 (IQR 30–90) from 60 (IQR 15–75), though most remained dependent.

By 3 months, recovery progressed: median NIHSS fell to 2 (IQR 1.5–10.5), mRS to 2 (IQR 1–5.25), and Barthel Index to 95 (IQR 10–100), indicating increased independence. At 1 year, gains persisted, with median NIHSS at 2 (IQR 0–19.5), mRS at 1 (IQR 0–6) (over 50% of survivors achieved mRS 0–2), and Barthel Index at 95 (IQR 2.5–100), showing near-complete recovery in many.

Statistical significance was confirmed with Friedman tests: NIHSS (χ^2^(3) = 41.6, *p* < 0.001), mRS (χ^2^(3) = 32.6, *p* < 0.001), and Barthel Index (χ^2^(3) = 44.3, *p* < 0.001), indicating a robust recovery trajectory despite missing data at later time points.

### 3.3. Associations Between Perfusion and Inflammation–Coagulation Indices and Functional Outcomes

**Figure 2:**
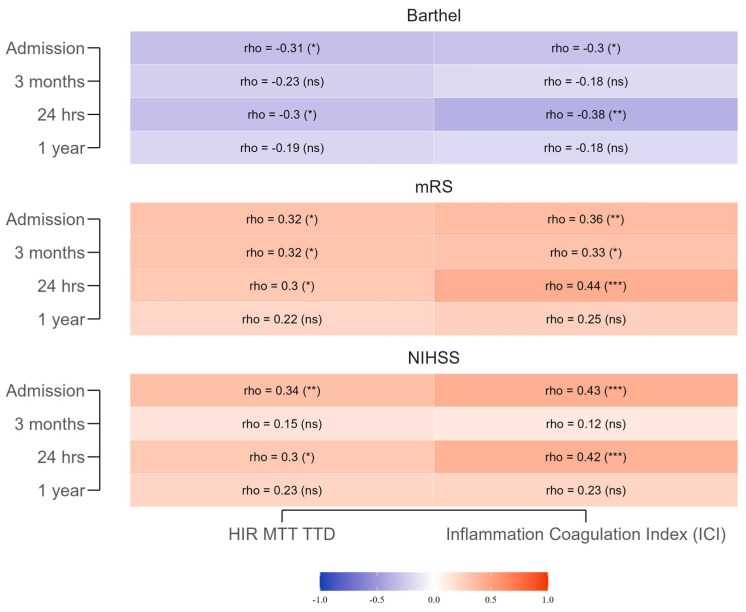
Spearman correlations between the HIR-MTT–TTD Index, the Inflammation–Coagulation Index, and functional outcomes (NIHSS, mRS, Barthel Index) across different time points. The gradient scale represents the strength and direction of the correlation coefficients: warmer shades of red indicate stronger positive correlations, while cooler shades of blue indicate stronger negative correlations. *** for *p* < 0.001, ** for *p* < 0.01, * for *p* < 0.05, and (ns) for non-significant (*p* ≥ 0.05).

Significant correlations emerged between the HIR-MTT–TTD index and early outcomes (Figure 2). Moderate positive correlations were noted with NIHSS at admission (r = 0.342, *p* = 0.008) and 24 h (r = 0.298, *p* = 0.021), indicating that higher HIR values were associated with greater neurological impairment, consistent with more severe perfusion deficits. These associations weakened at 3 months (r = 0.153, *p* = 0.304) and 1 year (r = 0.227, *p* = 0.133), suggesting that the early impact of hypoperfusion on neurological status diminishes over time as other recovery factors take precedence. Negative correlations with Barthel Index were observed at admission (r = −0.309, *p* = 0.016) and 24 h (r = −0.302, *p* = 0.019), reflecting poorer functional independence in patients with higher HIR values; these, too, were not significant at later time points (3 months: r = −0.226, *p* = 0.127; 1 year: r = −0.193, *p* = 0.204). Positive correlations with mRS were significant at admission (r = 0.325, *p* = 0.011) and 24 h (r = 0.300, *p* = 0.020), indicating greater disability in those with more pronounced hypoperfusion, but these lost statistical significance by 1 year.

The ICI showed stronger, more consistent early correlations. At admission, positive correlations with NIHSS (r = 0.429, *p* = 0.001) and mRS (r = 0.364, *p* = 0.004) and a negative correlation with Barthel Index (r = −0.296, *p* = 0.022) indicated that heightened systemic inflammation and coagulation activation were linked to greater stroke severity and reduced functional independence. These patterns persisted at 24 h (NIHSS: r = 0.417, *p* = 0.001; mRS: r = 0.437, *p* < 0.001; Barthel Index: r = −0.378, *p* = 0.003), supporting their role as early prognostic indicators. By 3 months and 1 year, correlations weakened and lost significance, reflecting a reduced influence of initial inflammatory status on long-term outcomes. Clinically, these results suggest that both indices have value for short-term prognostication, with ICI showing slightly greater early predictive strength, but neither maintains robust predictive value beyond the acute phase.

### 3.4. Regression Analysis of Long-Term Outcomes

**Figure 3:**
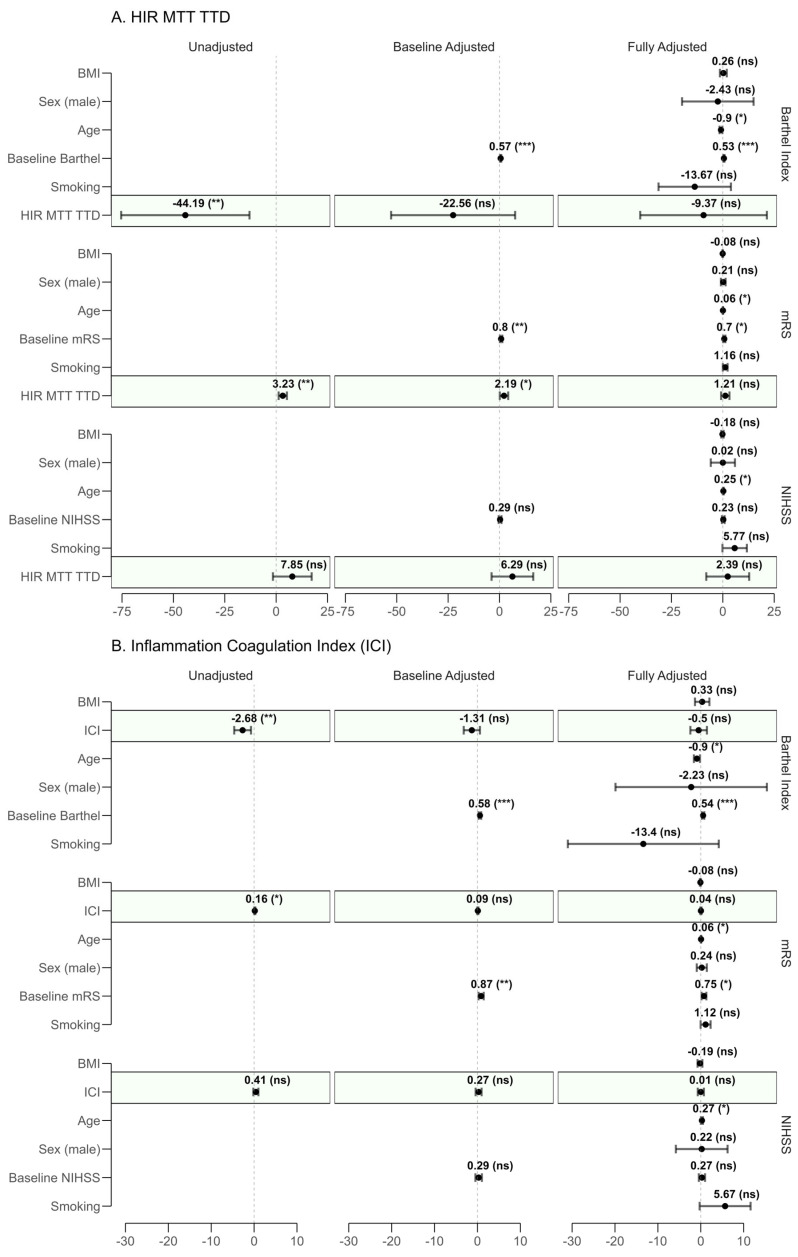
Forest plot of regression coefficients for (**A**) HIR-MTT–TTD and (**B**) Inflammation–Coagulation Index (ICI) across unadjusted, baseline-adjusted, and fully adjusted models. Dots represent the regression coefficients (β) with 95% confidence intervals (CI). The outcomes assessed are the last recorded modified Rankin Scale (mRS, where higher scores indicate worse disability), National Institutes of Health Stroke Scale (NIHSS, where higher scores indicate worse neurological status), and Barthel Index (where lower scores indicate worse independence). Each panel displays coefficients for relevant covariates (e.g., age, baseline mRS, baseline NIHSS, baseline Barthel Index, BMI, smoking status, and sex). Statistical significance is denoted as follows: *** for *p* < 0.001, ** for *p* < 0.01, * for *p* < 0.05, and (ns) for non-significant (*p* ≥ 0.05). A dashed vertical line at β = 0 serves as a reference for no effect. Shaded regions (green) in panels highlight the predictor of interest.

Linear regression models were fit to assess the predictive impact of HIR-MTT–TTD and ICI on the final observed record on long-term functional outcomes. Results from unadjusted, baseline-adjusted, fully adjusted, and fully adjusted combined models are detailed in Figure 3.

In unadjusted models, both HIR-MTT–TTD (β = 3.23, 95% CI: 1.22–5.23, *p* = 0.002) and ICI (β = 0.16, 95% CI: 0.04–0.29, *p* = 0.013) were significantly associated with increased disability. These associations persisted after baseline adjustment (HIR-MTT–TTD: *p* = 0.035; ICI: *p* = 0.170), but lost significance in the fully adjusted models (HIR-MTT–TTD: *p* = 0.247; ICI: *p* = 0.580). Notably, age emerged as a significant confounder (HIR-MTT–TTD: *p* = 0.021; ICI: *p* = 0.016).

No significant associations were observed in the prediction of NIHSS for either proposed predictor across unadjusted (HIR-MTT–TTD: *p* = 0.108; ICI: *p* = 0.175), baseline-adjusted (HIR-MTT–TTD: *p* = 0.229; ICI: *p* = 0.444), or fully adjusted models (HIR-MTT–TTD: *p* = 0.655; ICI: *p* = 0.979). However, age was significantly associated with higher NIHSS scores in the fully adjusted model (*p* = 0.038), suggesting a potential role in worse neurological outcomes.

For the Barthel Index, unadjusted models revealed significant negative associations with HIR-MTT–TTD (β = −44.19, 95% CI −75.42 to −12.96, *p* = 0.006) and ICI (β = −2.68, 95% CI −4.63 to −0.72, *p* = 0.008), indicating reduced independence. These became non-significant in fully adjusted models (HIR-MTT–TTD: *p* = 0.554; ICI: *p* = 0.617).

### 3.5. Survival Analysis

Over the 12-month follow-up, 14 out of 60 patients (23.3%) died, as confirmed by hospital records. Kaplan–Meier survival analysis estimated a survival probability of 84.9% (95% CI: 76.2–94.5%) at 30 days, which decreased to 79.8% (95% CI: 70.2–90.7%) at 90 days, further reduced to 76.4% (95% CI: 66.3–88.0%) at 180 days, and remained stable at 76.4% (95% CI: 66.3–88.0%) at 1 year, reflecting no additional deaths beyond 180 days.

To investigate factors linked to mortality, separate Cox proportional hazards models were developed for the HIR-MTT–TTD and Inflammation–Coagulation Index. The unadjusted model for HIR-MTT–TTD demonstrated a significant association with increased mortality risk (HR = 6.25, 95% CI: 1.48–26.30, *p* = 0.013), indicating that greater hypoperfusion severity and poorer collateral circulation were predictive of reduced survival. Similarly, the unadjusted model for ICI showed a significant association with mortality (HR = 1.08, 95% CI: 1.00–1.17, *p* = 0.052), suggesting that a heightened inflammatory and hypercoagulable state at admission elevated death risk, though this was borderline significant.

In multivariable models adjusted for age, BMI, smoking status, and sex, the associations attenuated. For HIR-MTT–TTD, the adjusted HR was 2.83 (95% CI: 0.37–21.37, *p* = 0.314), losing significance, while BMI emerged as a significant protective factor (HR = 0.80, 95% CI: 0.67–0.95, *p* = 0.011). For ICI, the adjusted HR was 1.07 (95% CI: 0.96–1.19, *p* = 0.211), also non-significant, with BMI again significant (HR = 0.78, 95% CI: 0.64–0.93, *p* = 0.008), indicating a protective effect against mortality.

**Figure 4:**
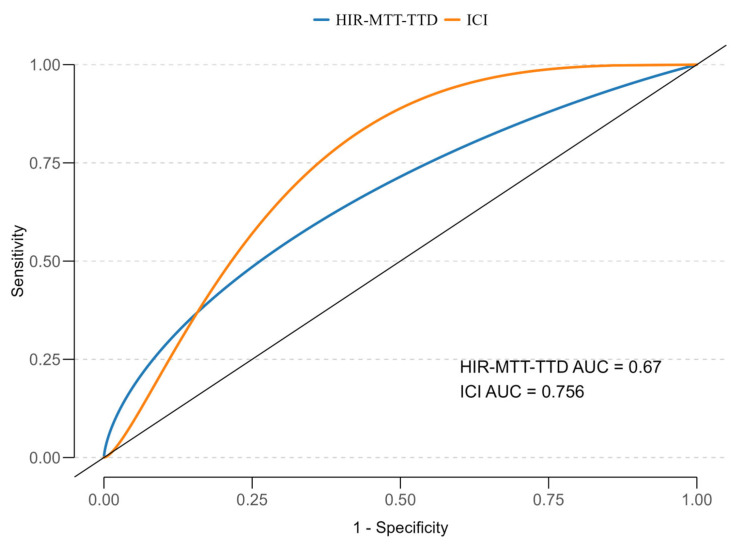
ROC curves for HIR-MTT–TTD and ICI in predicting 12-month mortality, with AUC values of 0.664 and 0.746, respectively.

To assess the discriminatory power of HIR-MTT–TTD and ICI for predicting mortality, ROC analysis with smoothed curves was performed (Figure 4). The AUC for HIR-MTT–TTD was 0.67 (95% CI: 0.48–0.83), and for ICI, it was 0.756 (95% CI: 0.600–0.867), both indicating moderate predictive ability. ICI demonstrated slightly better discrimination compared to HIR-MTT–TTD, though neither reached excellent predictive thresholds.

## 4. Discussion

### 4.1. Interpretation of HIR-MTT–TTD

Our results indicate that the novel perfusion index HIR-MTT–TTD (hypoperfusion intensity ratio derived from MTT and TTD maps) strongly correlates with both initial stroke severity and long-term outcomes. Patients with higher HIR-MTT–TTD values had more severe neurological deficits on admission (higher NIHSS scores) and experienced worse functional recovery, as reflected by higher mRS scores, lower Barthel Index scores, and increased 1-year mortality rates. This aligns with the known prognostic significance of the conventional Tmax-based HIR. Prior studies using RAPID software to compute HIR (ratio of volume with Tmax > 10 s to volume with Tmax > 6 s) have shown that a low HIR correlates with better collaterals, smaller core infarcts, and ultimately good functional outcomes [33,34,35]. Conversely, an elevated HIR signifies a large fraction of critically hypoperfused tissue (poor collaterals), which has been linked to more severe strokes, faster infarct growth, and higher risks of disability [36]. Lyndon et al. showed that an HIR > 0.45 predicted poor collaterals with an area under the curve (AUC) of 0.86, correlating strongly with computed tomography angiography (CTA) collateral grades (r = −0.55, *p* < 0.001) in patients with large vessel occlusion (LVO) [37]. In a more recent study, Miller et al. evaluated 231 patients with acute ischemic stroke and found that HIR ≥ 0.54 was independently associated with higher NIHSS scores at 24 h and significantly increased the risk of early neurological deterioration (OR 5.26, 95% CI 1.17–23.67, *p* = 0.03), particularly among patients with LVO [38]. These data further support the clinical relevance of HIR as a predictor of neurological decline even in patients who undergo reperfusion therapies. Moreover, HIR has been linked to the risk of hemorrhagic transformation. In a study by You et al. that included 168 patients treated with successful thrombectomy, every 0.1 increase in HIR was associated with 68% higher odds of hemorrhagic transformation (adjusted OR 1.68, 95% CI 1.26–2.25, *p* < 0.001), with an AUC of 0.80 for predicting parenchymal hematoma [39].

Our findings extend these observations: using MTT and TTD to calculate HIR, we similarly found that an unfavorable ratio corresponds to extensive perfusion delays and poor clinical prognosis.

The rationale for introducing MTT and TTD into the HIR calculation is to enhance sensitivity to the full spectrum of perfusion dynamics. Tmax measures the delay to peak contrast arrival (after deconvolution), which is useful but might not capture prolonged tissue circulation times or outflow resistance [40]. In contrast, MTT reflects the average time blood spends in the capillary bed, and TTD is the time from arterial arrival to contrast washout, effectively measuring how long perfusion remains in the tissue. TTD is a deconvolution-based parameter sensitive to hemodynamic disturbances not accounted for by Tmax or simple delay maps. By incorporating TTD (alongside MTT), HIR-MTT–TTD is attuned to sluggish microvascular flow and collateral circulation efficiency. In practical terms, a high HIR-MTT–TTD signifies that a large portion of the at-risk tissue has markedly prolonged transit and clearance times—an imaging signature of poor collaterals and impending infarction. This could explain its robust correlation with outcomes, as severe perfusion delays often presage larger final infarcts and complications like edema. Moreover, TTD and MTT have demonstrated excellent reproducibility in whole-brain CT perfusion analyses [41], supporting their reliability for quantitative indices. In summary, our HIR-MTT–TTD behaves analogously to the established Tmax-based HIR. Patients with lower ratios had milder deficits and better recovery, whereas those with high ratios fared worse, but it potentially captures perfusion failure more comprehensively by leveraging parameters (MTT, TTD) that reflect both capillary transit and venous outflow. This more nuanced assessment of cerebral hypoperfusion may provide a sensitive imaging biomarker of collateral status and ischemic burden beyond the traditional Tmax delay approach.

The observed associations between both HIR-MTT–TTD and ICI with mortality and functional outcomes were significant in unadjusted models but attenuated after adjustment for age, BMI, smoking status, and sex. This attenuation likely reflects the influence of these covariates as established prognostic factors in acute ischemic stroke. In particular, BMI emerged as a significant protective factor in our cohort, consistent with the “obesity paradox” [42] described in stroke outcomes literature, which may have overshadowed the independent effect of the studied indices. Additionally, partial collinearity between the indices and covariates—such as age-related differences in collateral status or inflammation—may have reduced the apparent independent predictive value of HIR-MTT–TTD and ICI after multivariable adjustment. These findings suggest that while the indices capture relevant pathophysiological processes, their prognostic contribution is interlinked with other major clinical predictors.

From a practical perspective, a higher HIR-MTT–TTD at baseline CTP flags patients with extensive microvascular delay. This information can support early triage decisions: prioritizing direct-to-angiography activation when LVO is suspected or CTA findings are equivocal; favoring expedited or extended-window reperfusion strategies in centers where logistics are borderline; and anticipating edema growth and allocating higher-intensity monitoring (ICU vs. stroke unit). Conversely, a low HIR-MTT–TTD suggests slower infarct progression and more effective collateral flow, which may justify IVT-first strategies with close clinical–imaging surveillance when endovascular access is delayed. HIR-MTT–TTD should be used as an adjunct to NIHSS, CTA/CTP core–penumbra metrics, and time-to-reperfusion, not as a stand-alone eligibility criterion.

### 4.2. Prognostic Value of ICI

We also found that the ICI, integrating C-reactive protein, D-dimer, and white blood cell count, holds significant prognostic value in acute ischemic stroke. Higher ICI values, reflecting enhanced thrombo-inflammatory activity relative to reduced immune response, correlated with greater stroke severity (higher NIHSS), increased disability (higher mRS), and higher 1-year mortality. This is biologically plausible; CRP, an acute-phase reactant, links to severe strokes and poor prognosis, with Idicula et al. noting higher NIHSS and mortality with elevated CRP [43]. D-dimer, a fibrin degradation product, predicts larger infarcts and poor outcomes [44], supported by a meta-analysis showing increased 3-month mortality risk with high levels [45]. Elevated WBC, indicating inflammation, is tied to worse outcomes and recurrent events [46], with a 10-year study linking admission leukocytosis to higher mortality. ICI’s composite nature (CRP + D-dimer/WBC) captures this synergy, where a high ratio may signal excessive inflammatory–thrombotic drive with limited immune reserve, common in severe strokes.

This aligns with the literature showing the additive prognostic power of combined markers. Peng et al. found patients with high D-dimer (>1.1 µg/mL) and leukocytosis had a six-fold higher in-hospital mortality hazard [47], while the China National Stroke Registry reported doubled 1-year disability odds with elevated hs-CRP, D-dimer, and lipoprotein(a) [33]. Unlike prior indices like neutrophil-to-lymphocyte ratio (NLR) [34], ICI uniquely merges inflammation (CRP), coagulation (D-dimer), and immune response (WBC), offering a novel systemic risk summary. A high ICI may identify patients with large infarcts and compromised immunity, vulnerable to complications. These findings support ICI as a promising prognostic tool, warranting comparison with existing indices like NLR in future studies.

ICI can be calculated from standard admission labs within minutes. A high ICI identifies patients at heightened systemic risk and may justify intensified bedside care; more frequent neurological checks, early dysphagia screening, vigilant temperature and glucose control, optimization of VTE prophylaxis, and early rehabilitation input. In thrombolysed patients, the combination of high ICI with high HIR-MTT–TTD delineates a very-high-risk phenotype, prompting closer surveillance for complications. Conversely, a low ICI is useful for patient-family counselling about a greater likelihood of favorable recovery.

### 4.3. Strengths and Limitations

The introduction of two novel indices—HIR-MTT–TTD, integrating advanced neuroimaging markers of perfusion and collateral status, and ICI, summarizing key inflammatory and coagulative biomarkers—offers an innovative, interdisciplinary approach for capturing the complex pathophysiology of acute ischemic stroke. To minimize potential biases, all imaging analyses were performed using a uniform acquisition protocol and processed on the same software platform, with evaluations conducted by trained neurologists blinded to laboratory results and long-term outcomes. Standardized clinical assessments (NIHSS, mRS, Barthel Index) were used to reduce inter-rater variability. Despite these measures, heterogeneity in outcomes may still arise from unmeasured variables such as differences in collateral circulation quality, microvascular integrity, systemic inflammatory response, time-to-reperfusion, and comorbidity burden, which were not fully captured in this dataset. A comprehensive evaluation of neurological improvement (NIHSS), functional status (mRS, Barthel Index), and mortality allowed for a nuanced understanding of prognostic potential.

However, several limitations should be acknowledged. The observational design precludes causal inference and increases susceptibility to residual confounding, particularly by unmeasured or poorly captured clinical factors. The small sample size (n = 60) limits statistical power, increasing the risk of both type II error and unstable estimates, particularly in multivariable models, and constrains the ability to conduct stratified or interaction analyses. As a single-center study, the findings may be influenced by center-specific practices and case mix, which restricts external validity and limits generalizability beyond IVT-treated patients, particularly to those undergoing endovascular therapy or managed in different healthcare systems.

Although our findings suggest that HIR-MTT–TTD and ICI have potential prognostic utility, the present study did not include direct comparisons with established indices, such as the conventional Tmax-based hypoperfusion intensity ratio or hematological markers like the neutrophil-to-lymphocyte ratio (NLR) [48]. For NLR, differential leukocyte counts were not consistently available for all patients, and computation of traditional HIR would require reprocessing perfusion datasets using alternative thresholds. As a result, we were unable to formally evaluate whether the proposed indices outperform these established measures or quantify their incremental predictive value. Future studies with larger, multicenter cohorts and harmonized imaging and laboratory protocols are warranted to conduct head-to-head comparisons, ideally integrating these metrics into multivariable prognostic models. Such analyses would help determine whether HIR-MTT–TTD and ICI provide an additive benefit over existing tools in guiding early risk stratification and treatment decisions in acute ischemic stroke.

Additional methodological concerns include the single-time-point measurement of ICI, which may not reflect the dynamic nature of systemic inflammation or coagulation changes during the acute and subacute phases of stroke. The HIR-MTT–TTD metric, while promising, is dependent on vendor-specific perfusion software, raising potential challenges in reproducibility across institutions and imaging platforms. Information bias may arise from imperfect measurement of perfusion parameters or laboratory markers, while misclassification of exposure or outcome variables—especially in the context of retrospective NIHSS scoring or incomplete clinical data—could attenuate observed associations. Furthermore, the relatively low number of mortality events constrains the precision of survival estimates and the robustness of Cox regression findings.

Variability in outcomes among patients with similar baseline HIR-MTT–TTD or ICI values may be explained by additional pathophysiological and clinical factors not fully captured in our models. Collateral circulation quality plays a pivotal role in sustaining perfusion and delaying infarct progression, and patients with well-developed collaterals may demonstrate favorable outcomes despite high perfusion delay metrics [49]. Conversely, impaired microvascular integrity or a heightened systemic inflammatory and procoagulant response may accelerate tissue injury and worsen prognosis even when perfusion parameters appear relatively preserved. Other contributors include differences in time-to-reperfusion, the extent of core–penumbra mismatch, and baseline comorbidity burden, all of which can influence both acute neurological status and long-term recovery. These interacting factors may modulate the prognostic strength of HIR-MTT–TTD and ICI, underscoring the need for integrated, multimodal assessment in acute ischemic stroke.

Finally, the HIR-MTT–TTD metric, while promising, is dependent on vendor-specific perfusion software (in this study, Siemens syngo.via), which may limit reproducibility across centers using different platforms. Differences in deconvolution algorithms, perfusion map generation, and threshold application could result in variability of calculated indices. To enhance broader applicability, future research should explore cross-vendor validation, establish consensus on perfusion parameter definitions and thresholds, and consider developing open-source or vendor-neutral processing pipelines to standardize HIR-MTT–TTD calculation across diverse clinical environments.

Taken together, these limitations underscore the need for larger, multicenter validation studies with standardized imaging and biomarker protocols, longitudinal sampling, and more diverse patient populations. The present results should be considered hypothesis-generating, providing a basis for further investigation rather than definitive conclusions.

## 5. Conclusions

This prospective study of 60 thrombolysis-treated acute ischemic stroke patients introduced HIR-MTT–TTD and ICI as novel prognostic indices. HIR-MTT–TTD, derived from CT perfusion (MTT and TTD), correlated lower values with better recovery and higher values with poor collaterals and worse outcomes. ICI, integrating CRP, D-dimer, and WBC, linked elevated levels to increased severity and poorer functional recovery, highlighting the role of thrombo-inflammation.

These findings highlight the potential clinical utility of HIR-MTT–TTD and ICI as easy-to-obtain prognostic tools in acute stroke care. The HIR-MTT–TTD can be readily calculated from standard CT perfusion scans to gauge collateral flow quality, providing rapid quantitative insight that could impact treatment decisions and risk stratification beyond conventional imaging metrics. Likewise, the ICI is derived from routine blood tests and may help flag patients at increased risk of unfavorable outcomes, enabling more personalized monitoring and adjunctive therapies.

As this is an initial single-center study with a relatively small sample size, the findings should be interpreted with caution. The limited cohort and potential center-specific factors may restrict generalizability to broader stroke populations, particularly those undergoing different reperfusion strategies or managed in varied healthcare settings. Therefore, further validation of these indices in larger, prospective, multicenter cohorts is essential to confirm their prognostic value and refine their integration into clinical workflows. Future research should also explore their role in guiding patient selection for aggressive reperfusion therapies and in tailoring post-acute management strategies.

## Figures and Tables

**Table 1:** Baseline characteristics of the study population by mortality outcome at the end of the study period.

Characteristic	Overall N = 60	Alive N = 46	Death N = 14	*p* Value
Age ^1^	72 (62, 79)	70 (60, 76)	85 (72, 91)	<0.01
Sex, male ^2^	30 (50%)	24 (52%)	6 (43%)	0.5
BMI ^1,3^	25.7 (21.7, 29.9)	26.8 (23.7, 31.6)	20.6 (18.6, 25.7)	<0.01
Systolic blood pressure ^1,4^	140 (120, 160)	140 (130, 170)	140 (120, 140)	0.2
Diastolic blood pressure ^1,4^	80 (75, 100)	80 (80, 100)	80 (70, 90)	0.3
Active smokers ^3^	24 (40%)	18 (39%)	6 (43%)	0.8
Stroke type ^3^				
WUS	11 (18%)	7 (15%)	4 (29%)	0.3
SUO	5 (8.3%)	2 (4.3%)	3 (21%)	0.08
ETW	14 (23%)	10 (22%)	4 (29%)	0.7
Comorbidities ^3^				
Diabetes	44 (73%)	33 (72%)	11 (79%)	0.7
Pre-existing CVD	58 (97%)	44 (96%)	14 (100%)	>0.9
MTT penumbra volume ^1,5^	20 (14, 25)	19 (15, 25)	20 (13, 24)	0.5
MTT infarct volume ^1,5^	5.0 (3.1, 8.0)	4.6 (2.9, 7.1)	6.3 (4.0, 11.0)	0.06
TTD penumbra volume ^1,5^	20 (14, 25)	19 (15, 25)	20 (13, 24)	0.5
TTD infarct volume ^1,5^	5.3 (3.2, 8.3)	5.0 (3.0, 7.8)	6.3 (4.0, 11.0)	0.09
CRP ^1,6^	12 (5, 44)	10 (4, 21)	35 (16, 56)	0.01
D-dimer ^1,7^	1.6 (0.5, 4.6)	1.1 (0.5, 3.2)	4.7 (2.0, 9.8)	0.01
WBC ^1,8^	8.76 (7.12, 11.85)	8.76 (7.12, 11.39)	8.86 (7.23, 12.40)	0.8

^1^ Median (Q1, Q3); ^2^ Number (%) of individuals; ^3^ BMI—Body mass index in kg/m^2^; ^4^ Systolic and diastolic blood pressure in mmHg; ^5^ Volumes in milliliters (mL), derived from perfusion imaging; ^6^ C-reactive protein (CRP) in mg/L; ^7^ D-dimer in mg/L FEU; ^8^ WBC—White blood cell count (in scale of 10^9^/L); CVD—Cardiovascular diseases; WUS—Wake-up stroke; SUO—Stroke of unknown onset; ETW—Extended therapeutic window.

## Data Availability

The data supporting the findings of this study are available from the corresponding author upon reasonable request. However, due to privacy and ethical restrictions, raw patient data are not publicly available.

## Supplementary Materials

The following supporting information can be downloaded at: https://www.mdpi.com/article/10.3390/neurolint17090136/s1, Supplementary Materials: Reproducibility Appendix and Synthetic Data.

## Abbreviations

The following abbreviations are used in this manuscript:

HIR	Hypoperfusion intensity ratio
CTP	Computed tomography perfusion
ICI	Inflammation and coagulation index
CRP	C-reactive protein
WBC	White blood count
NIHSS	National Institutes of Health Stroke Scale
mRS	Modified Rankin Scale
CT	Computed tomography
WUS	Wake-up stroke
SUKO	Stroke of unknown onset
ETW	Extended therapeutic window
AHA/ASA	The American Heart Association/American Stroke Association
DWI-MRI	Diffusion-weighted magnetic resonance imaging
rCBF	Relative cerebral blood flow
MTT	Mean transit time
TTD	Time-to-drain
Tmax	Time-to-maximum
IVT	Intravenous thrombolysis
CBF	Cerebral blood flow
CBV	Cerebral blood volume
HR	Hazard ratio
CI	Confidence interval
AIC	Akaike Information Criterion
ROC	Receiver operating characteristic
AUC	Area under the curve
BMI	Body mass index
IQR	Interquartile range
CVD	Cardiovascular disease
CTA	Computed tomography angiography
LVO	Large vessel occlusion
NLR	Neutrophil-to-lymphocyte ratio
