# Supplementary material for: Novel Computed Tomography Perfusion and Laboratory Indices as Predictors of Long-Term Outcome and Survival in Acute Ischemic Stroke
Source: Neurol Int. 2025 Aug 27;17(9):136. doi: 10.3390/neurolint17090136 (PMC12472316; doi:10.3390/neurolint17090136)
Supplement: Supplementary file 1 [file neurolint-17-00136-s001.zip › neurolint-3792081-supplementary.pdf]

Reproducibility Appendix and Synthetic Data

Novel Computed Tomography Perfusion and Laboratory Indi-ces as Predictors of Long-Term Outcome and Survival in Acute Ischemic Stroke

Table of contents

1 S1. Data availability and reproducibility 1
2 S2. Software environment 2
3 S3. Simulated cohort 2
4 S4. Descriptive summaries (Table 1) 5
5 S5. Outcome distributions 5
6 S6. Correlations 6
7 S7. Linear models for functional outcomes 7
8 S8. Survival analysis 9
9 S9. ROC curves with smoothing 11
10 S10. Notes on missing data handling and coding 12
11 S11. Session info 12

S1. Data availability and reproducibility

Patient-level data contain personal health information and cannot be shared under the study's ethics approval and GDPR. To ensure reproducibility, this appendix provides a **synthetic dataset** with the same structure and plausible value ranges as the original cohort, together with the **exact analysis workflow**. All outputs herein are derived from simulated data and are for methodological illustration only.

> ⓘ Important
>
> This document is fully self-contained. Running it will: 1) Simulate a cohort of 60 patients.
> 2) Produce descriptive summaries, correlation plots, linear models, survival models (with PH checks), and smoothed ROC curves.
> 3) Render figures directly in the document.

S2. Software environment

```
req_pkgs <- c(
  "dplyr","tidyr","forcats","ggplot2","broom","survival","pROC",
  "ggcorrplot","patchwork","gtsummary","Hmisc"
)

to_install <- req_pkgs[!suppressWarnings(sapply(req_pkgs, requireNamespace,
quietly = TRUE))]
if (length(to_install)) install.packages(to_install, repos =
"https://cloud.r-project.org")
invisible(lapply(req_pkgs, library, character.only = TRUE))

set.seed(20250814)
```

S3. Simulated cohort

We simulate 60 patients with variables mirroring the manuscript (higher **HIR-MTT–TTD** and **ICI** indicate worse pathophysiology). Follow-up and mortality risks are tied to indices and age to approximate observed relationships.

```
n <- 60
id <- 1:n

age  <- round(rnorm(n, mean = 76, sd = 9))
male <- rbinom(n, 1, 0.48)
bmi  <- round(rnorm(n, mean = 25.5, sd = 3.8), 1)
smoking <- rbinom(n, 1, 0.35)

# Indices
hir_mtt_ttd <- pmax(0, rbeta(n, 2.2, 3.0)) * 1.5        # ~0-1.5
inflamation_coagulation_index_ici <- round(rlnorm(n, 0.9, 0.5), 2)

# Baseline outcomes
nihss_admision <- pmin(42,
  round( 6 + 9*hir_mtt_ttd + 0.25*inflamation_coagulation_index_ici +
rnorm(n,0,3) )
)
nihss_admision[nihss_admision < 0] <- 0

m_rs_admision <- pmin(6,
  pmax(0, round( 2 + 1.6*hir_mtt_ttd + 0.06*inflamation_coagulation_index_ici
+ rnorm(n,0,1) ))
)

barthel_index_admision <- pmin(100,
  pmax(0, round( 55 - 15*hir_mtt_ttd - 1.5*inflamation_coagulation_index_ici
+ rnorm(n,0,12) ))
)
```

```r
# Follow-ups (average improvement)
nihss_24hrs <- pmax(0, round(nihss_admision - rnorm(n, mean = 2, sd = 2)))
nihss_3mo   <- pmax(0, round(nihss_24hrs - rnorm(n, mean = 2, sd = 2)))
nihss_1yr   <- pmax(0, round(nihss_3mo   - rnorm(n, mean = 1, sd = 2)))

m_rs_24hrs <- pmin(6, pmax(0, round(m_rs_admision - rnorm(n, mean = 0.3, sd =
0.8))))
m_rs_3mo   <- pmin(6, pmax(0, round(m_rs_24hrs - rnorm(n, mean = 0.4, sd =
0.9))))
m_rs_1yr   <- pmin(6, pmax(0, round(m_rs_3mo   - rnorm(n, mean = 0.2, sd =
0.8))))

barthel_index_24hrs <- pmin(100, pmax(0, round(barthel_index_admision +
rnorm(n, 5, 10))))
barthel_index_3mo   <- pmin(100, pmax(0, round(barthel_index_24hrs +
rnorm(n,15,12))))
barthel_index_1yr   <- pmin(100, pmax(0, round(barthel_index_3mo   + rnorm(n,
5,10))))

# Survival & follow-up
follow_up_days <- round(runif(n, min=60, max=365))
linpred <- 0.6*hir_mtt_ttd + 0.05*inflamation_coagulation_index_ici +
0.015*(age-75) - 0.04*(bmi-25)
risk <- plogis(linpred - 1.2) # ~20–30% events
death <- rbinom(n, 1, risk)

# "Last known" outcomes (illustrative; real data followed ethics rules)
last_known_nihss <- ifelse(death==1, nihss_24hrs, ifelse(!is.na(nihss_1yr),
nihss_1yr, nihss_3mo))
last_known_barthel <- ifelse(death==1, barthel_index_24hrs,
ifelse(!is.na(barthel_index_1yr), barthel_index_1yr, barthel_index_3mo))
last_known_m_rs <- ifelse(death==1, 6, ifelse(!is.na(m_rs_1yr), m_rs_1yr,
m_rs_3mo))

dat <- tibble(
  id, age, male, bmi, smoking,
  hir_mtt_ttd, inflamation_coagulation_index_ici,
  nihss_admision, nihss_24hrs, nihss_3mo, nihss_1yr,
  m_rs_admision, m_rs_24hrs, m_rs_3mo, m_rs_1yr,
  barthel_index_admision, barthel_index_24hrs, barthel_index_3mo,
barthel_index_1yr,
  follow_up_days, death,
  last_known_nihss, last_known_barthel, last_known_m_rs
)

glimpse(dat)
```

```
Rows: 60
Columns: 24
$ id                                  <int> 1, 2, 3, 4, 5, 6, 7, 8, 9, 10, 11,
1…
$ age                                 <dbl> 84, 62, 84, 71, 72, 77, 68, 77, 70,
…
$ male                                <int> 1, 0, 0, 0, 1, 0, 1, 0, 0, 0, 0, 0,
…
$ bmi                                 <dbl> 24.3, 23.9, 24.3, 28.0, 24.2, 21.7,
…
$ smoking                             <int> 0, 0, 0, 1, 1, 0, 1, 0, 0, 0, 0, 0,
…
$ hir_mtt_ttd                         <dbl> 1.0521607, 0.6036132, 0.4521869,
0.6…
$ inflamation_coagulation_index_ici <dbl> 1.44, 3.89, 2.40, 6.17, 2.35, 6.87,
…
$ nihss_admision                      <dbl> 13, 13, 9, 15, 7, 14, 10, 18, 15,
15…
$ nihss_24hrs                         <dbl> 11, 9, 8, 14, 8, 12, 11, 14, 11,
13,…
$ nihss_3mo                           <dbl> 7, 4, 7, 9, 6, 7, 8, 12, 7, 13, 7,
1…
$ nihss_1yr                           <dbl> 2, 0, 7, 8, 4, 8, 6, 13, 7, 12, 7,
1…
$ m_rs_admision                       <dbl> 2, 3, 4, 4, 3, 3, 3, 4, 4, 6, 2, 3,
…
$ m_rs_24hrs                          <dbl> 2, 3, 4, 4, 2, 4, 3, 4, 6, 6, 2, 5,
…
$ m_rs_3mo                            <dbl> 4, 1, 3, 4, 2, 5, 2, 5, 6, 6, 1, 4,
…
$ m_rs_1yr                            <dbl> 2, 1, 2, 4, 2, 4, 1, 6, 6, 4, 0, 4,
…
$ barthel_index_admision              <dbl> 25, 41, 43, 33, 53, 27, 53, 47, 32,
…
$ barthel_index_24hrs                 <dbl> 34, 22, 52, 38, 42, 39, 50, 54, 42,
…
$ barthel_index_3mo                   <dbl> 53, 47, 66, 70, 48, 63, 60, 89, 40,
…
$ barthel_index_1yr                   <dbl> 43, 64, 76, 85, 75, 78, 64, 92, 43,
…
$ follow_up_days                      <dbl> 342, 136, 103, 121, 258, 197, 171,
7…
$ death                               <int> 0, 0, 0, 0, 0, 0, 0, 0, 0, 0, 0, 0,
…
$ last_known_nihss                    <dbl> 2, 0, 7, 8, 4, 8, 6, 13, 7, 12, 7,
1…
$ last_known_barthel                  <dbl> 43, 64, 76, 85, 75, 78, 64, 92, 43,
…
$ last_known_m_rs                     <dbl> 2, 1, 2, 4, 2, 4, 1, 6, 6, 4, 0, 4,
…
```

S4. Descriptive summaries (Table 1)

```
tbl1 <- dat %>%
  select(death, age, male, bmi, smoking, hir_mtt_ttd,
inflamation_coagulation_index_ici) %>%
  gtsummary::tbl_summary(by = death, missing = "no") %>%
  add_overall() %>%
  add_p()
tbl1
```

**Characteristic**	**Overall**, N = 60[1]	**0**, N = 43[1]	**1**, N = 17[1]	**p-value**[2]
age	75 (70, 82)	75 (70, 83)	75 (70, 81)	0.8
male	24 (40%)	15 (35%)	9 (53%)	0.2
bmi	26.2 (24.0, 28.7)	26.0 (24.3, 28.6)	26.3 (21.8, 30.0)	0.5
smoking	16 (27%)	15 (35%)	1 (5.9%)	0.025
hir_mtt_ttd	0.58 (0.43, 0.82)	0.56 (0.45, 0.82)	0.63 (0.38, 0.77)	0.7
inflamation_coagulation_index_ici	2.55 (1.91, 3.77)	2.91 (2.16, 3.81)	2.14 (1.76, 3.06)	0.085

[1]Median (IQR); n (%)

[2]Wilcoxon rank sum test; Pearson's Chi-squared test; Fisher's exact test; Wilcoxon rank sum exact test

5 S5. Outcome distributions

```
mk_long <- function(df, prefix, pretty){
  df %>%
    select(id, starts_with(prefix)) %>%
    pivot_longer(-id, names_to="tp", values_to="value") %>%
    mutate(tp = factor(tp,
      levels = paste0(prefix, c("_admision","_24hrs","_3mo","_1yr")),
      labels = c("Admission","24 hrs","3 months","1 year")),
      outcome = pretty)
}

p_mrs <- mk_long(dat, "m_rs", "mRS") %>%
  ggplot(aes(x = value, y = tp, fill = after_stat(x))) +
  ggdist::stat_slab(aes(thickness = after_stat(pdf*n)), side="both",
slab_linewidth=NA) +
  scale_fill_viridis_c() + labs(x="mRS", y=NULL) +
theme(legend.position="none")

p_bi  <- mk_long(dat, "barthel_index", "Barthel") %>%
  ggplot(aes(x = value, y = tp, fill = after_stat(x))) +
  ggdist::stat_slab(aes(thickness = after_stat(pdf*n)), side="both",
slab_linewidth=NA) +
  scale_fill_viridis_c() + labs(x="Barthel Index", y=NULL) +
```

```
theme(legend.position="none")

p_nih <- mk_long(dat, "nihss", "NIHSS") %>%
  ggplot(aes(x = value, y = tp, fill = after_stat(x))) +
  ggdist::stat_slab(aes(thickness = after_stat(pdf*n)), side="both",
slab_linewidth=NA) +
  scale_fill_viridis_c() + labs(x="NIHSS", y=NULL) +
theme(legend.position="none")

(p_mrs + p_bi + p_nih) + patchwork::plot_layout(nrow = 1)
```

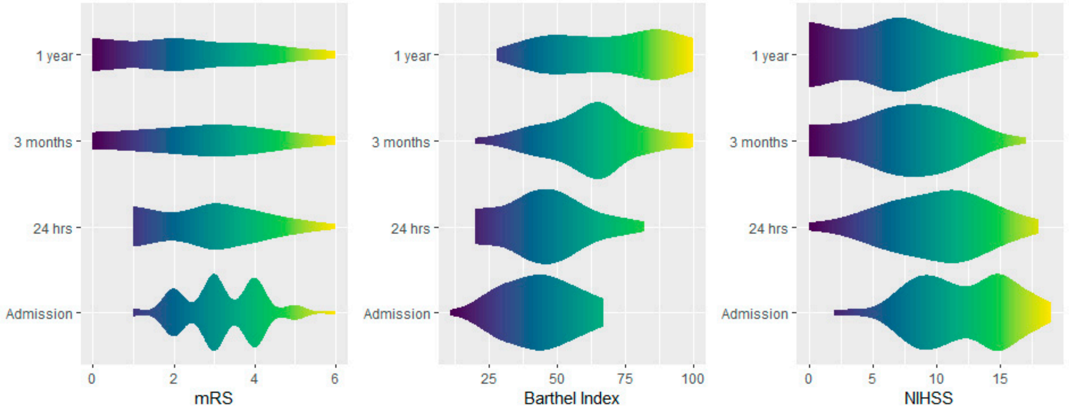

Distributions of mRS, Barthel, and NIHSS across time points (synthetic data).

S6. Correlations

```
keep <- c("nihss_admision","barthel_index_admision","m_rs_admision",
          "nihss_24hrs","barthel_index_24hrs","m_rs_24hrs",
          "nihss_3mo","barthel_index_3mo","m_rs_3mo",
          "nihss_1yr","barthel_index_1yr","m_rs_1yr")

rc <- Hmisc::rcorr(as.matrix(dat[, keep]), type = "spearman")
ggcorrplot::ggcorrplot(rc$r, type = "lower", lab = TRUE) + labs(x=NULL,
y=NULL)
```

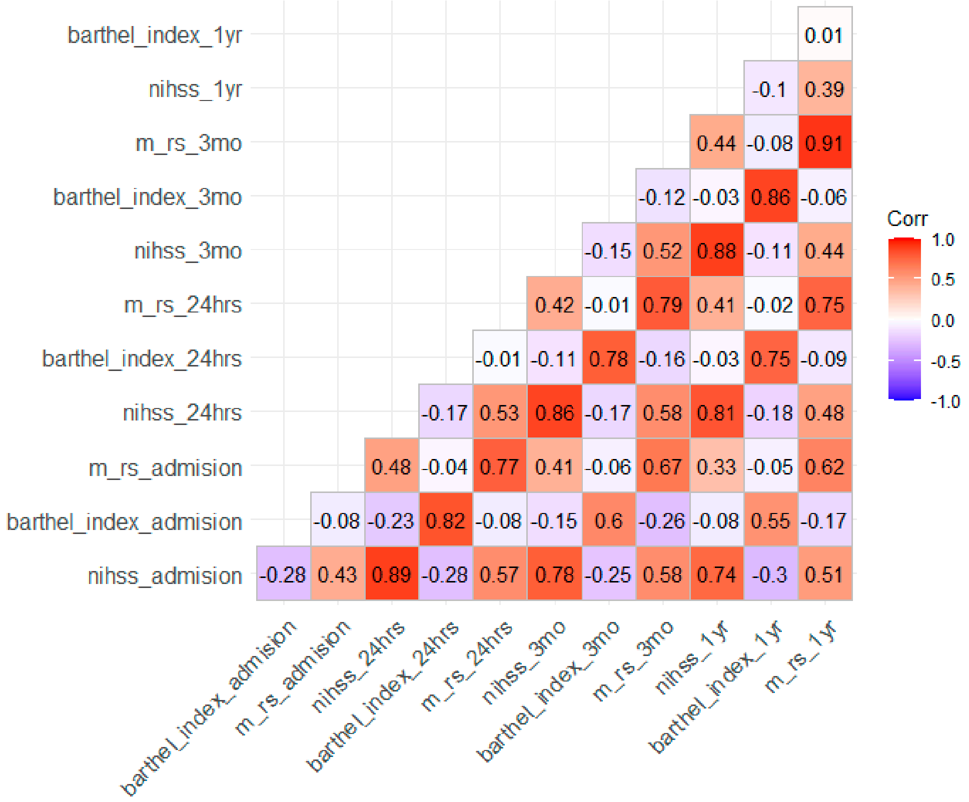

Spearman correlations between outcomes at each time point (synthetic data).

S7. Linear models for functional outcomes

We fit unadjusted, baseline-adjusted, and fully adjusted models for mRS, NIHSS, and Barthel "last known" scores with **HIR-MTT–TTD** and **ICI** as predictors.

```
to_model <- list(
  list(outcome="last_known_m_rs", base="m_rs_admision"),
  list(outcome="last_known_nihss", base="nihss_admision"),
  list(outcome="last_known_barthel", base="barthel_index_admision")
)

fit_one <- function(outcome, base, predictor, type=c("unadj","base","full")){
  type <- match.arg(type)
  if (type=="unadj"){
    as.formula(paste0(outcome," ~ ", predictor))
  } else if (type=="base"){
    as.formula(paste0(outcome," ~ ", predictor," + ",base))
  } else {
    as.formula(paste0(outcome," ~ ", predictor," + ",base," + age + bmi +
smoking + male"))
  }
}
```

```r
rows <- lapply(to_model, function(m){
  lapply(c("hir_mtt_ttd","inflamation_coagulation_index_ici"),
function(pred){
    lapply(c("unadj","base","full"), function(tp){
      fm <- fit_one(m$outcome, m$base, pred, tp)
      mod <- lm(fm, data=dat)
      broom::tidy(mod) |>
        mutate(outcome = m$outcome, predictor = pred, type = tp,
               lower = estimate - 1.96*std.error,
               upper = estimate + 1.96*std.error)
    }) |> bind_rows()
  }) |> bind_rows()
}) |> bind_rows()

forest_hir <- rows %>% filter(predictor=="hir_mtt_ttd", term!="(Intercept)")
ggplot(forest_hir, aes(x=estimate, y=forcats::fct_reorder(term, estimate))) +
  geom_point() +
  geom_errorbarh(aes(xmin=lower, xmax=upper), height=0.2) +
  facet_grid(outcome ~ type) +
  geom_vline(xintercept=0, linetype="dashed", color="grey50") +
  labs(x=NULL, y=NULL, title="A. HIR-MTT-TTD")
```

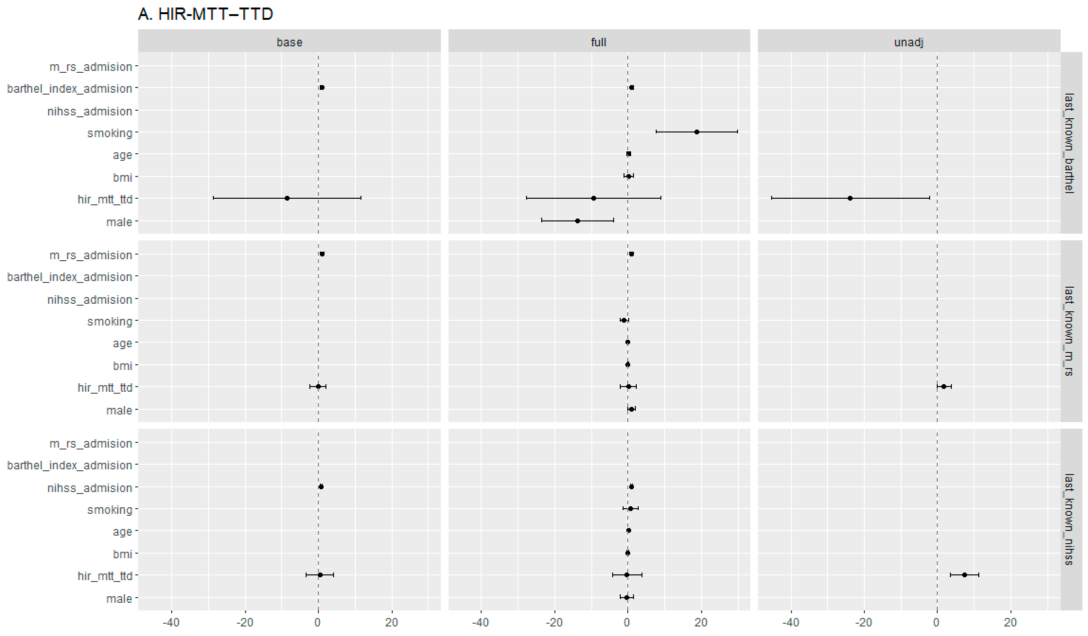

Estimated effects and 95% CIs for predictors across model types (synthetic data).

```r
forest_ici <- rows %>% filter(predictor=="inflamation_coagulation_index_ici",
term!="(Intercept)")
ggplot(forest_ici, aes(x=estimate, y=forcats::fct_reorder(term, estimate))) +
  geom_point() +
  geom_errorbarh(aes(xmin=lower, xmax=upper), height=0.2) +
  facet_grid(outcome ~ type) +
```

```
  geom_vline(xintercept=0, linetype="dashed", color="grey50") +
  labs(x=NULL, y=NULL, title="B. Inflammation–Coagulation Index (ICI)")
```

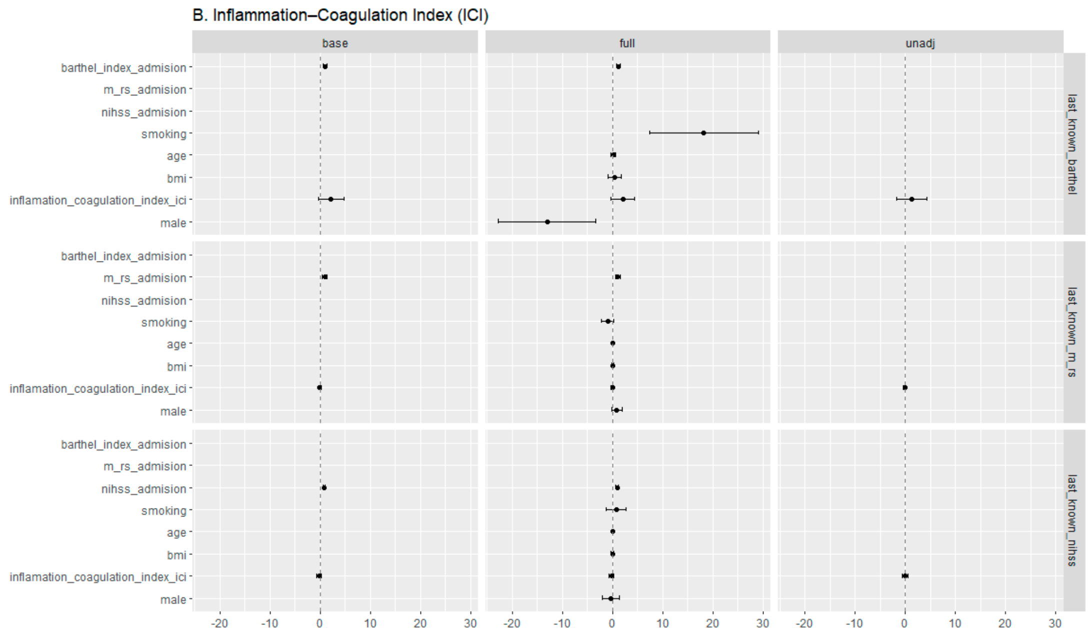

Estimated effects and 95% CIs for predictors across model types (synthetic data).

S8. Survival analysis

We estimate Kaplan–Meier survival and fit Cox models (unadjusted and adjusted). PH assumptions are checked via Schoenfeld residuals.

```
surv_obj <- Surv(time = dat$follow_up_days, event = dat$death)
km_fit <- survfit(surv_obj ~ 1)
summary(km_fit, times = c(30, 90, 180, 365))

Call: survfit(formula = surv_obj ~ 1)

 time n.risk n.event survival std.err lower 95% CI upper 95% CI
   30     60       0    1.000   0.000        1.000        1.000
   90     57       0    1.000   0.000        1.000        1.000
  180     41       4    0.919   0.039        0.846        0.999

cox_hir <- coxph(surv_obj ~ hir_mtt_ttd, data = dat)
cox_hir_adj <- coxph(surv_obj ~ hir_mtt_ttd + age + bmi + smoking + male,
data = dat)
cox_ici <- coxph(surv_obj ~ inflamation_coagulation_index_ici, data = dat)
cox_ici_adj <- coxph(surv_obj ~ inflamation_coagulation_index_ici + age + bmi
+ smoking + male, data = dat)

broom::tidy(cox_hir, exponentiate = TRUE, conf.int = TRUE)
```

```
# A tibble: 1 × 7
  term        estimate std.error statistic p.value conf.low conf.high
  <chr>          <dbl>     <dbl>     <dbl>   <dbl>    <dbl>     <dbl>
1 hir_mtt_ttd    0.570     0.981    -0.573   0.567   0.0835      3.90

broom::tidy(cox_hir_adj, exponentiate = TRUE, conf.int = TRUE)

# A tibble: 5 × 7
  term        estimate std.error statistic p.value conf.low conf.high
  <chr>          <dbl>     <dbl>     <dbl>   <dbl>    <dbl>     <dbl>
1 hir_mtt_ttd    0.540    0.952     -0.648  0.517    0.0835      3.49
2 age            1.03     0.0306     1.00   0.317    0.971       1.09
3 bmi            0.948    0.0795    -0.672  0.502    0.811       1.11
4 smoking        0.128    1.06      -1.95   0.0515   0.0161      1.01
5 male           1.80     0.502      1.17   0.242    0.673       4.81

broom::tidy(cox_ici, exponentiate = TRUE, conf.int = TRUE)

# A tibble: 1 × 7
  term                      estimate std.error statistic p.value conf.low
conf.high
  <chr>                        <dbl>     <dbl>     <dbl>   <dbl>    <dbl>
<dbl>
1 inflamation_coagulati…    0.867     0.188    -0.763   0.445    0.600
1.25

broom::tidy(cox_ici_adj, exponentiate = TRUE, conf.int = TRUE)

# A tibble: 5 × 7
  term                      estimate std.error statistic p.value conf.low
conf.high
  <chr>                        <dbl>     <dbl>     <dbl>   <dbl>    <dbl>
<dbl>
1 inflamation_coagulati…    0.844    0.203     -0.837  0.402    0.567
1.26
2 age                       1.03     0.0300     0.927  0.354    0.969
1.09
3 bmi                       0.968    0.0762    -0.426  0.670    0.834
1.12
4 smoking                   0.132    1.04      -1.94   0.0520   0.0172
1.02
5 male                      1.63     0.499      0.975  0.329    0.612
4.33

# PH checks
cox.zph(cox_hir)

            chisq df    p
hir_mtt_ttd  0.33  1 0.57
GLOBAL       0.33  1 0.57

cox.zph(cox_hir_adj)
```

```
              chisq df    p
hir_mtt_ttd 0.19347  1 0.66
age         1.08868  1 0.30
bmi         0.06257  1 0.80
smoking     2.04185  1 0.15
male        0.00666  1 0.93
GLOBAL      4.94618  5 0.42
```

```
cox.zph(cox_ici)
```

```
                                   chisq df   p
inflamation_coagulation_index_ici 0.0662  1 0.8
GLOBAL                            0.0662  1 0.8
```

```
cox.zph(cox_ici_adj)
```

```
                                     chisq df    p
inflamation_coagulation_index_ici 0.014254  1 0.90
age                               1.461401  1 0.23
bmi                               0.249330  1 0.62
smoking                           2.006483  1 0.16
male                              0.000693  1 0.98
GLOBAL                            4.522276  5 0.48
```

9 S9. ROC curves with smoothing

AUCs and 95% CIs are computed using binormal smoothing. Curves are plotted for both indices.

```
roc_hir <- pROC::roc(dat$death, dat$hir_mtt_ttd, ci=TRUE, smooth=TRUE)
roc_ici <- pROC::roc(dat$death, dat$inflamation_coagulation_index_ici,
ci=TRUE, smooth=TRUE)

cat("HIR-MTT-TTD AUC:", pROC::auc(roc_hir), "  95% CI:",
pROC::ci.auc(roc_hir), "\n")
```

```
HIR-MTT-TTD AUC: 0.4657267   95% CI: 0.3038451 0.4623786 0.6197148
```

```
cat("ICI AUC:", pROC::auc(roc_ici), "  95% CI:", pROC::ci.auc(roc_ici), "\n")
```

```
ICI AUC: 0.6340874   95% CI: 0.4706801 0.6302004 0.7776378
```

```
coords_hir <- as.data.frame(coords(roc_hir, x="all", input="sensitivity",
                                   ret=c("sensitivity","specificity")))
coords_ici <- as.data.frame(coords(roc_ici, x="all", input="sensitivity",
                                   ret=c("sensitivity","specificity")))

plot_df <- bind_rows(
  mutate(coords_hir, Index = "HIR-MTT-TTD"),
  mutate(coords_ici, Index = "ICI")
)
```

```r
ggplot(plot_df, aes(x = 1-specificity, y = sensitivity, color = Index)) +
  geom_line(linewidth = 1.2) +
  geom_abline(slope = 1, intercept = 0) +
  labs(x = "1 - Specificity", y = "Sensitivity", title = NULL) +
  theme(legend.position = "top")
```

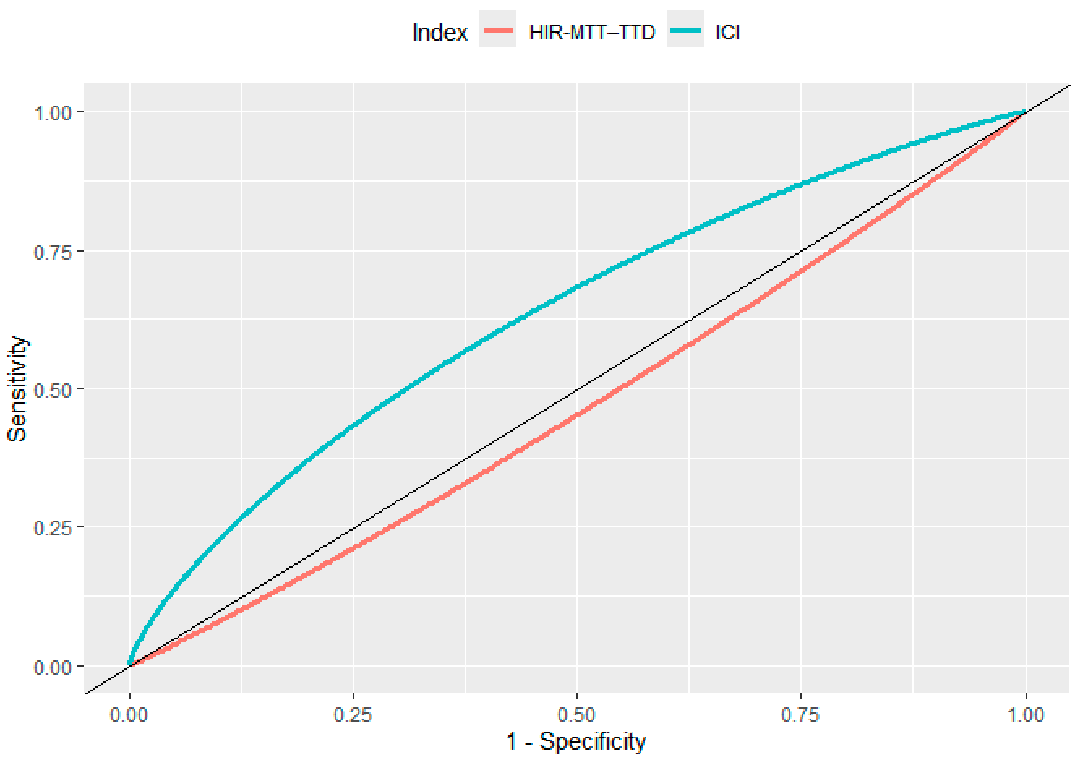

Smoothed ROC curves for 12-month mortality (synthetic data).

S10. Notes on missing data handling and coding

Primary analyses use complete cases. If a patient dies before a planned follow-up, mRS at the next time point is coded as 6. NIHSS and Barthel Index are **not** imputed with extreme values; the last observed scores are retained. For analyses requiring a single outcome value, we derive a "last known" outcome variable.

S11. Session info

```r
sessionInfo()

R version 4.3.3 (2024-02-29 ucrt)
Platform: x86_64-w64-mingw32/x64 (64-bit)
Running under: Windows 11 x64 (build 22631)

Matrix products: default
```

```
locale:
[1] LC_COLLATE=English_United States.utf8
[2] LC_CTYPE=English_United States.utf8
[3] LC_MONETARY=English_United States.utf8
[4] LC_NUMERIC=C
[5] LC_TIME=English_United States.utf8

time zone: Europe/Sofia
tzcode source: internal

attached base packages:
[1] stats     graphics  grDevices utils     datasets  methods   base

other attached packages:
 [1] Hmisc_5.1-2        gtsummary_1.7.2    patchwork_1.2.0
ggcorrplot_0.1.4.1
 [5] pROC_1.18.5        survival_3.8-3     broom_1.0.5        ggplot2_3.5.1
 [9] forcats_1.0.0      tidyr_1.3.1        dplyr_1.1.4

loaded via a namespace (and not attached):
 [1] tidyselect_1.2.1       viridisLite_0.4.2      farver_2.1.1
 [4] fastmap_1.1.1          fontquiver_0.2.1       promises_1.3.0
 [7] broom.helpers_1.15.0   digest_0.6.35          rpart_4.1.23
[10] mime_0.12              lifecycle_1.0.4        cluster_2.1.6
[13] ellipsis_0.3.2         gfonts_0.2.0           magrittr_2.0.3
[16] compiler_4.3.3         rlang_1.1.3            tools_4.3.3
[19] utf8_1.2.4             yaml_2.3.8             gt_0.10.1
[22] data.table_1.17.8      knitr_1.46             labeling_0.4.3
[25] askpass_1.2.0          htmlwidgets_1.6.4      curl_5.2.1
[28] plyr_1.8.9             xml2_1.3.6             httpcode_0.3.0
[31] withr_3.0.0            foreign_0.8-86         purrr_1.0.2
[34] nnet_7.3-19            grid_4.3.3             fansi_1.0.6
[37] gdtools_0.3.7          xtable_1.8-4           colorspace_2.1-0
[40] scales_1.3.0           crul_1.4.2             cli_3.6.2
[43] rmarkdown_2.26         crayon_1.5.2           ragg_1.3.0
[46] generics_0.1.3         rstudioapi_0.16.0      reshape2_1.4.4
[49] stringr_1.5.1          splines_4.3.3          base64enc_0.1-3
[52] vctrs_0.6.5            Matrix_1.6-5           jsonlite_1.8.8
[55] fontBitstreamVera_0.1.1 Formula_1.2-5         htmlTable_2.4.2
[58] systemfonts_1.0.6      ggdist_3.3.2           glue_1.7.0
[61] distributional_0.4.0   stringi_1.8.3          flextable_0.9.5
[64] gtable_0.3.5           later_1.3.2            munsell_0.5.1
[67] tibble_3.2.1           pillar_1.9.0           htmltools_0.5.8.1
[70] openssl_2.1.2          R6_2.5.1               textshaping_0.3.7
[73] evaluate_0.23          shiny_1.8.1.1          lattice_0.22-5
[76] backports_1.4.1        fontLiberation_0.1.0   httpuv_1.6.15
[79] zip_2.3.1              uuid_1.2-0             Rcpp_1.0.12
```

```
[82] gridExtra_2.3          checkmate_2.3.1        officer_0.6.5
[85] xfun_0.43              pkgconfig_2.0.3
```
